# Spasmogenic and spasmolytic activity of rind of *Punica granatum* Linn

**DOI:** 10.1186/s12906-017-1616-4

**Published:** 2017-02-07

**Authors:** Niaz Ali, Ayesha Jamil, Syed Wadood Ali Shah, Ismail Shah, Ghayour Ahmed

**Affiliations:** 1 Department of Pharmacology, Institute of Basic Medical Sciences, Khyber Medical University, Peshawar, KPK Pakistan; 2 Department of Pharmacology, Khyber Girls Medical College, Hayatabad, Peshawar, Khyber Pakhtunkhwa Pakistan; 3 Department of Pharmacy, University of Malakand, Chakdara, Dir, Khyber Pakhtunkhwa Pakistan; 4 Department of Pharmacy, The Abdul Wali Khan University, Mardan, Khyber Pakhtunkhwa Pakistan; 5 Assistant Director, Drug Regulatory Authority of Pakistan, Islamabad, Pakistan

**Keywords:** *Punica granatum*, Calcium channel blocker, Spasmogenic, Spasmolytic, Verapamil

## Abstract

**Background:**

Rind of *Punica granatum* is traditionally used in treatment of abdominal cramps and various GIT disorders. So far spasmolytic activity of rind of *Punica granatum* has been reported using in vitro model. However, its mode of action is not explored yet. Therefore, the current work describes the possible mode of action for spasmolytic activity of methanolic extract of rind of *Punica granatum* (Pg. Cr). Acute toxicity study is also performed to determine its safe dose range.

**Methods:**

Rind of *Punica granatum* was subjected to shade drying. Shade dried materials were pulverized using conventional grinder. Grinded materials were macerated in commercial grade methanol. The extract of rind of *P. granatum* was concentrated using a rotary evaporator. Rabbits’ jejunal preparations were mounted in organ bath containing 10 ml Tyrode’s solution, constantly aerated with carbogen gas. Pg. Cr was tested on spontaneous rabbits’ jejunal preparations in concentrations 0.01, 0.03, 0.1, 0.3, 1.0, 3.0, 5.0 and 10.0 mg/ml. Pg. Cr was also tested on KCl (80 mM)-induced contractions in rabbits’ jejunal preparations. Since we observed spasmogenic activity for the first time, hence we also determined the effects of Pg. Cr in presence of atropine (0.03 μM). Pg. Cr was also tested in presence of 0.03 μM of loratadine HCl. Pg. Cr was also tested on barium chloride induced contractions. Calcium Concentration Response Curves (CCRCs) were constructed in the absence and presence of test samples of Pg. Cr in decalcified tissues to explore its possible mode of action. Acute toxicity screening was also performed to determine its safe dose range.

**Results:**

Phytochemical screening revealed the presence of saponins, tannins, carbohydrates, proteins, flavonoids, saponins and steroids. However, Pg. Cr tested negative for alkaloids and triterpenoids. Pg. Cr was safe up to 100 mg/kg with its LD_50 =_ 1305 mg/kg. Its respective EC_50,_ in the absence and presence of atropine, were 9.7 ± 0.3 and 3.12 ± 0.45 mg/ml. In the presence of 0.02 and 0.08 μM of loratadine HCl, respective EC_50_ were 5.6 ± 0.4 and 2.8 ± 0.15 mg/ml. EC_50_ for relaxant effects on KCl-induced contractions was 8.6 ± 1 mg/ml. In the presence of 0.3 mg/ml of Pg. Cr, a right shift was observed with EC_50_ (log [Ca^++^]M) _=_ -1.8 ± 0.09 vs. control EC_50_ -2.6 ± 0.01. Similarly, EC_50_ for verapamil (0.1 μM) was −2.4 ± 0.011*vs.* control EC_50=_ -2.4 ± 0.01. The right shift of *P. granatum* resembled the right shift of verapamil suggesting for inhibition of voltage gated calcium channels.

**Conclusions:**

*P. granatum* is safe up to 100 mg/kg. In low concentrations, *P. granatum* produced spasmogenic activity possibly through involvement of cholinergic and histaminergic receptors. The spasmolytic action may follow inhibition of the voltage gated calcium channels.

## Background

Herbal medicine contributes to revenue of 2.5 $US in countries like Japan, China, Pakistan, Sri Lanka and Thailand [1, 2]. In general perspective, medicines of plant origin are considered relatively safe [3]. Plant kingdom is a rich source of bioactive molecules. For example morphine, strychnine, cinchonine, quinine and caffeine were first derived from plants [4]. With the passage of time, chemists started synthesis of these molecules in laboratories. Cocaine was synthesized by Schiff in laboratory [5]. According to a report of WHO, more than 50% of the world population depend on household remedies. Pharmacopoeia of the United States describes various phyto-medicines that are also used in Europe [6]. This strengthen the practice of complementary and alternative medicine for the maintenance of health in developing countries [7].

*P. granatum* is a plant that is referenced in Holy Quran by name. It belongs to the order of Myrtales and family of Lythraceace. More than 500 cultivators throughout the world cultivate *P. granatum* [8]. It is abundantly available in Himalayan regions and Kashmir. It grows at an altitude of 1000–2000 m in Northern built (South Waziristan, Khyber Pakhtun Khwa, Dir, Kurram, Chitral and Baluchistan) of Pakistan. It is used as medicine to treat obesity, arthritis and ischemia of brains [9]. More, *P. granatum* has anti-oxidant, anticancer and anti-inflammatory activities [10].

Resistant strains of Bacteria like *Staphylococcus aureus*, *Streptococcus epidermidis, Lactobacillus acidophilus* and *E.coli* have shown excellent sensitivity to *Punica granatum* [11]. It is also used for treatment of erectile dysfunctions [9]. Beside oral antiseptic, it is also used to treat periodontitis and dental carries [9]. *P. granatum* has anti-diabetic activity [8]. *P. granatum* is also used in treatment of skin infections and injuries, where its role may be attributed to its antioxidant effect [9, 10, 12]. Reported phytochemicals of *P. granatum* are anthocyanins, ascorbic acid, caffeic acid, catechin, Epigallocatechingallate (EGCG), quercetin, sterols, punicic acid, ellagic acid, phenolic punicalagins and flavonoids [9]. The juice of the seeds is rich in anthocyanins, ascorbic acid, EGCG and iron. The seed oil has 95% punicic acid, ellagic acid and sterols [9]. The leaves are rich in tannins (punicalin and punicafolin) and flavones glycosides, which include apigenin and luteolin [9]. The extract of leaves of *P. granatum* is used as eye washer, as astringent for diarrhea and dysentery [13]. The rind (peel) is rich in phenolic punicalagins, EGCG, quercetin, rutin, flavones, flavonols, flavonones, anthocyanidins gallic acid and other fatty acids [14]. There are reports that extract of dried rind (peel) of *P. granatum* is traditionally used in stomach ache and colitis. In Indian traditional system of medicine, *P. granatum* is used as an astringent, anthelmintic, diuretic and cardio tonic [11]. As peels of *P. granatum* is used in treatment of in gut spasms, therefore, the current work is an attempt to explore its rationale on scientific ground. More, there are studies that warrant for search of possible rationale for use of *P. granatum* in gut disorders to elucidate its mode of action [13]. Hence, we designed the current model to elucidate possible mode of action for spasmolytic activity of rind of *P. granatum.*

## Methods

### Collection, identification and preparation of the test materials

Fruits of *P. granatum* were purchased from the Board Bazar of Peshawar, Khyber Pakhtunkhawa. Professor Dr. Jehandar Shah identified the plant. A voucher specimen no. Pg-01 was submitted to the herbarium of Hakim Abdul Jalil Herbal Research Center, Khyber Medical University, Peshawar. Their fleshy seeds were removed. Its rind was collected and subjected to shade drying. We targeted the rind as it is locally used in the treatment of gastric disorders and diarrhea [13]. It is called “anarsawe” in Pashtoo. The dried rind (1.5 kg) was powdered using a conventional grinder. The powdered materials were then soaked in commercial grade methanol (80%) for 5 days. After 5 days, the materials were filtered. The process was repeated thrice. The filtrates were combined and concentrated under vacuum using a rotary evaporator till a brownish semisolid extract, free of solvent (20 g), was obtained. The extract was refrigerated for further pharmacological screenings.

### Preliminary phytochemical screenings

Preliminary phytochemical screenings were performed for the presence of tannins, carbohydrates, proteins, flavonoids, saponins, sterols, alkaloids and triterpenoids [15, 16].

### Acute toxicity study

Acute toxicity testing was performed using mice model. In first phase, Pg. Cr was administered in test doses of 10, 100 and 1000 mg/kg (i.p) to group 1, group 2 and group 3 respectively. Each group had six mice. In second phase, 3 more groups, having 6 six mice each, were treated with respective test doses of 1250, 1500 and 1750 mg/kg (i.p). Death toll was noted in each group. Per cent lethality was plotted against respective test doses. LD_50_ was calculated [17, 18].

### Drugs, chemicals, animals and ethical approval

All chemicals were of analytical grade. Acetylcholine was purchased from Poole England. Rest of the chemicals was of E. Merck grade, Germany. Double distilled water was used in the experiments. All solutions were prepared on the same day of experiments. For acute toxicity study, Pg. Cr was re-dissolved in water for injection (B.P.). Rabbits of either sex (average weight = 2.2 ± 0.24 kg) were purchased from the local market. Rabbits were housed in the animal house of Institute of Basic Medical Sciences, Khyber Medical University, Peshawar. Ethics Board of the Khyber Medical University in its 3rd meeting under agenda item no. 5 approved the study protocols on dated 8th April 2013. The protocols complied with international standards for dealing experimental animals for Scientific Procedures.

### Effects on Isolated rabbits’ jejunal preparations

#### On spontaneous rabbits’ jejunal preparations

Rabbits were subjected to cervical dislocation. Their abdomens were opened. Their jejunums were removed and maintained in Petri dishes containing Tyrode’s solution, constantly aerated with Carbogen gas (95% oxygen/5% carbon dioxide). Portions of about 1.5 cm length of rabbits’ jejunal preparations were mounted in tissue organ baths. The tissues were stabilized in normal Tyrode’s solution for about 30 min. Following stabilization, Pg. Cr was tested on isolated rabbits’ jejunal preparations in concentrations 0.01, 0.03, 0.1, 0.3, 1.0, 3.0, 5.0 and 10.0 mg/ml [19–24]. Changes in isometric tension were recorded using force transducers (model MLT0201) coupled with bridge amplifiers FE221 connected to PowerLab 26/T (ADInstruments, Sydney, Australia). Data was recorded using Lab Chart 7 software (ADInstruments, Sydney, Australia). Intestinal responses were plotted as % of control.

#### On spontaneous rabbits’ jejunal preparations in the presence of cholinergic antagonists (atropine) and histamine antagonist (loratadine)

Since Pg. Cr produced spasmogenic response in isolated rabbits’ jejunal preparations contrary to the study published by Qnais et al. [13]. Hence, we tried to explain the possible mode of spasmogenic action. As intestine is richly supplied with cholinergic and histaminergic receptors, therefore, we tried Pg. Cr in similar concentrations 0.01, 0.03, 0.1, 0.3, 1.0, 3.0, 5.0 and 10.0 mg/ml in presence of cholinergic antagonist atropine (0. 03 μM) following an incubation period of 20-25 min [25]. Pg. Cr was also tested in similar concentrations in presence of histamine H1 receptor antagonist loratadine HCl (0.02 and 0.08 μM) following an incubation period of 30 min. Intestinal responses were recorded using Lab Chart 7 Software.

#### Effects of Pg. Cr on KCl-induced contractions

As Pg. Cr showed relaxant effect in higher concentrations, hence we tried to determine its possible mode of relaxation. We tried Pg. Cr on KCl (80 mM) induced contractions. Briefly describing, small portions of the rabbits’ jejunal preparations were mounted in the organ baths. After stabilization, sustained contractions were produced by 80 mM KCl (final bath solution’s strength). Pg. Cr was applied in similar concentrations 0.01, 0.03, 0.1, 0.3, 1.0, 3.0, 5.0, 10.0 mg/ml. Intestinal responses were recorded [19].

#### Effect of Pg. Cr on barium chloride induced contractions

Sustained contractions in the rabbits’ jejunal preparations were produced by barium chloride (1.0 μM). Pg. Cr was added in similar concentrations 0.01, 0.03, 0.1, 0.3, 1.0, 3.0, 5.0, 10.0 mg/ml in cumulative manner. Effects were recorded [26].

#### Effects of verapamil of spontaneous and KCl-induced contractions

Rabbits’ jejunal preparations were mounted in tissue organ bath. After stabilizing the tissues for 30 min, verapamil (concentration range of 0.003–3 μM) was tested on spontaneous and KCl (80 mM)-induced contractions. Effects of test samples are compared with effects of verapamil as standard calcium channel blocker.

### Effects of Pg. Cr on voltage gated calcium channels

As Pg. Cr produced a spasmolytic response; hence we constructed calcium chloride curves to study its effects possibly through voltage gated channels as relaxant effects on high molar (>60 mM) KCl induced contractions usually, but not necessarily, imply to follow inhibition of voltage gated calcium channels. Hence, for confirmation, we compared the effects of Pg. Cr on calcium channels with the control Calcium Concentration Response Curves (CCRCs) using verapamil a standard calcium channel blocker.

For construction of CCRCs, the tissues were decalcified with the Potassium Normal Tyrode’s Solution (containing EDTA 0.03 g), followed by Potassium Rich Tyrode’s solution (containing K: 3.72 g and EDTA: 0.37 g). Once decalcified, known strengths of calcium were added and constructed the CCRCs. Curves were constructed in range of - 4 to - 1.6 log [Ca^++^] M.

In other series of experiments, tissues were stabilized in Tyrode’s solution for 30 min. The tissues were decalcified as described above. Pg. Cr was applied in different concentrations with an incubation period of one hour. CCRCs were reconstructed again in range of - 4 to - 1.6 log [Ca^++^] M. The process was repeated 4 times. The CCRCs for Pg. Cr were compared with its respective control. Similarly, control curves in presence of 0.1 μM and 0.3 μM of verapamil were also constructed. The CCRCs for verapamil were compared with its respective control curves [19, 21].

### Statistical analysis

All data extracted from Lab Chart of Power Lab was plotted for each replicate using Graph Pad Prism 7. The response of tissues was plotted as % of its respective control. Data was analyzed at 95% CI, *P* ≤ 0.05 using ANOVA.

## Results and discussion

Phytochemical screening revealed the presence of tannins, carbohydrates, proteins, flavonoids, saponins and steroids. However, Pg. Cr tested negative for alkaloids and triterpenoids. Test for the presence of saponins was strongly positive.

**Fig. 1:**
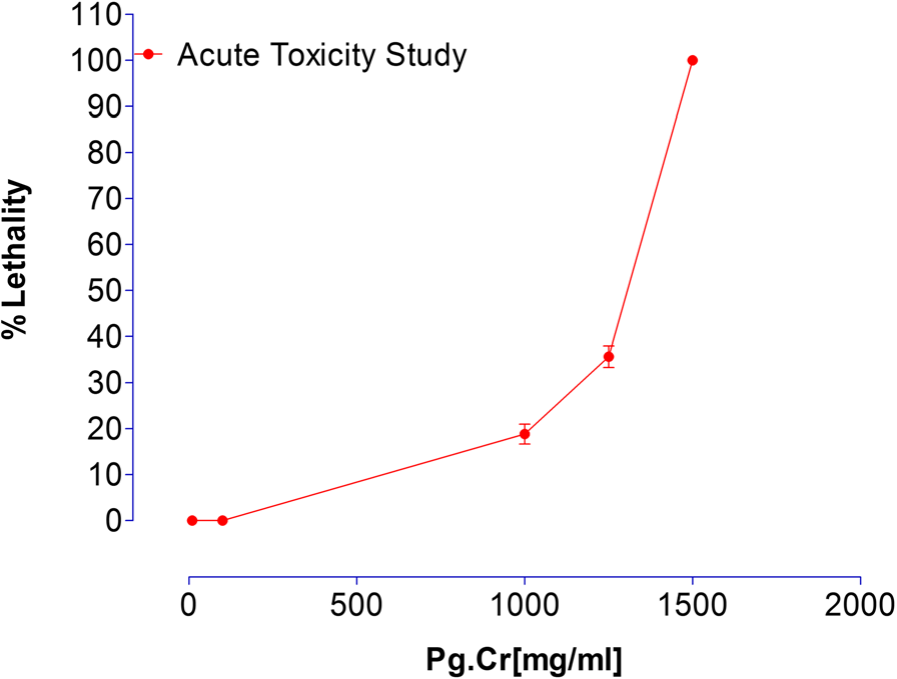
Acute toxicity study of crude methanolic extract of *Punica granatum* in mice

Table 1 describes the results of acute toxicity study. Results suggest that Pg. Cr is safe up to 100 mg/kg in test animals. Lethality started on 1000 mg/kg, where 2 of the total 6 mice in respective group died. Similarly, 2 mice died in group1 of 2^nd^ phase. Their % toxicity was plotted *vs*. respective doses. LD_50_ for Pg. Cr is 1305 mg/kg (Fig. 1).

**Table 1:** Results of acute toxicity of *Punica granatum* in mice

Stages	Dose (mg/kg body weight, (i.p)) (*n* = 6 in each group)		
1^st^ stage	Group1 (10 mg)	Group 2 (100 mg)	Group 3 (1000 mg)
	Alive	Alive	4 alive
2^nd^ stage	Group 1 (1250 mg)	Group 2 (1500 mg)	Group 3 (1750 mg)
	4 died	All died	All died

Hence, care must be taken while using its rind as traditional medicine. Its dose should not be more than 100 mg/kg as it carries risks of acute toxicity.

**Fig. 2:**
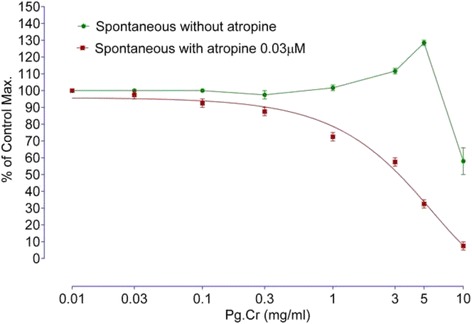
Effects of crude methanolic extract of *Punica granatum* on spontaneous rabbits’ jejunal preparations in the presence and absence of atropine (All values are mean ± SD, *n* = 3)

**Fig. 3:**
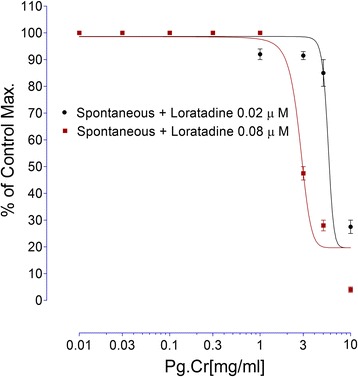
Effects of different concentrations of crude methanolic extract of *Punica granatum* in the presence of loratadine HCl (All values are mean ± SD, *n* = 3)

**Fig. 4:**
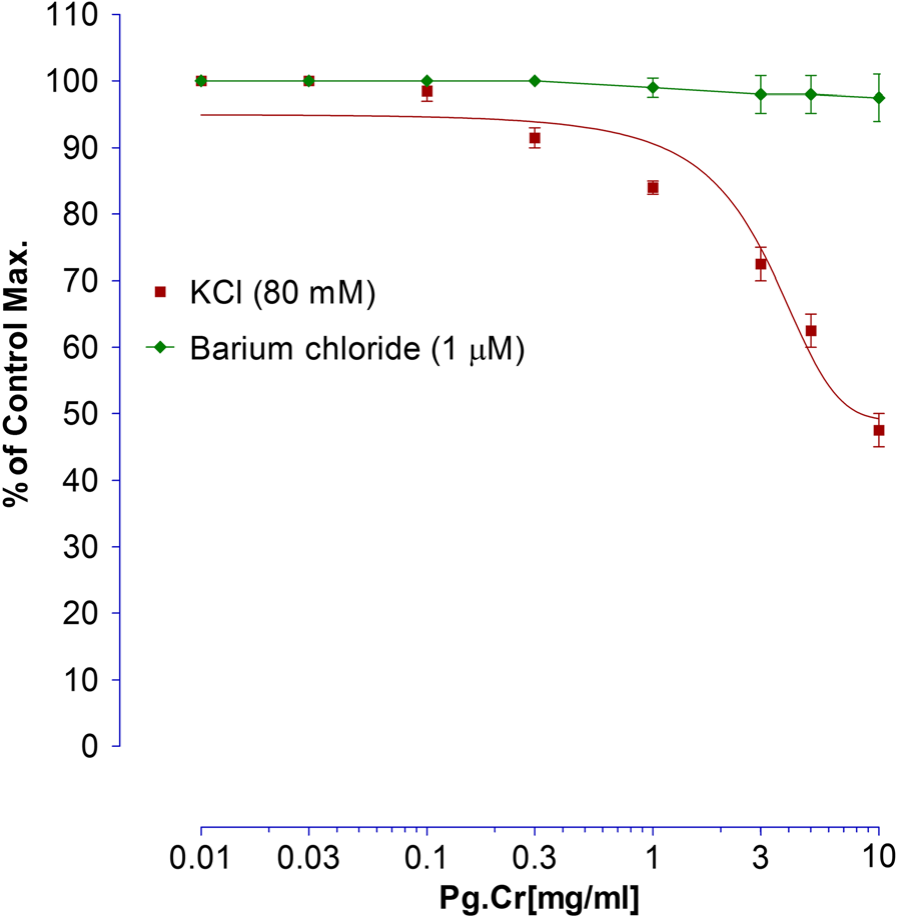
Effects of crude methanolic extract of *Punica granatum* on high concentration (80 mM) KCl-induced contractions on rabbits’ jejunal preparations and on Barium chloride induced contractions (All values are mean ± SD, *n* = 3)

**Fig. 5:**
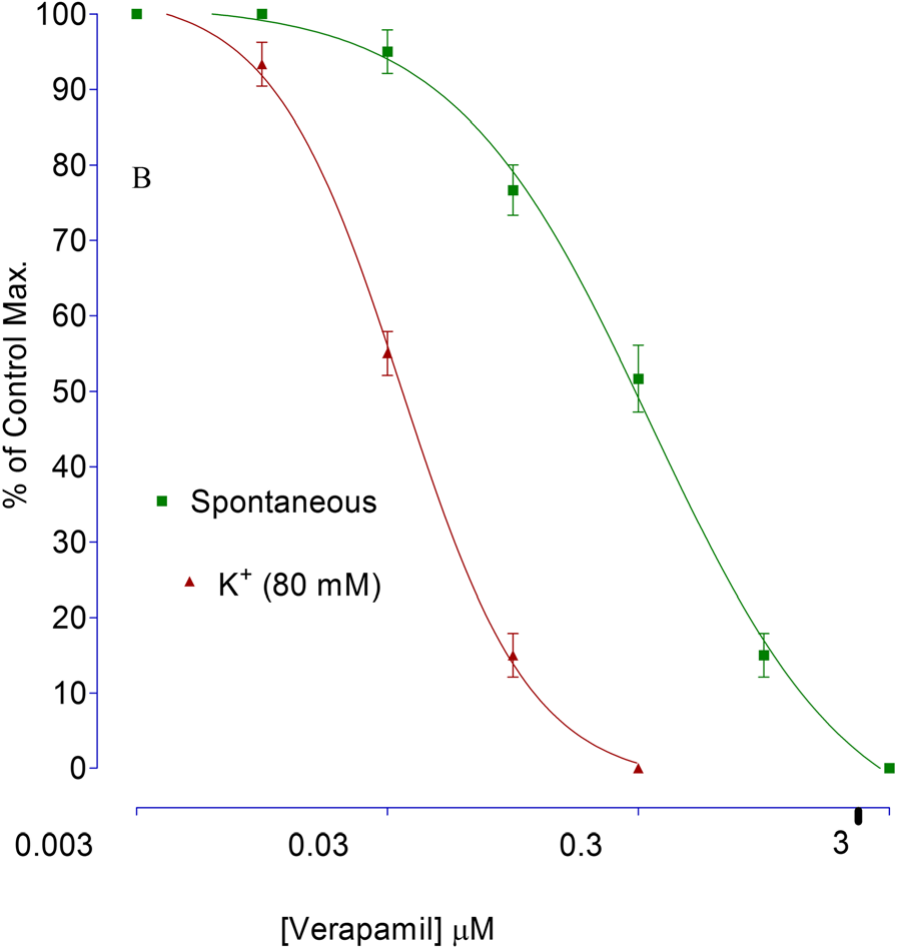
Effects of verapamil on spontaneous as well as KCl-induced contractions on isolated rabbits’ jejunal preparations (All values are mean ± SD, *n* = 3)

**Fig. 6:**
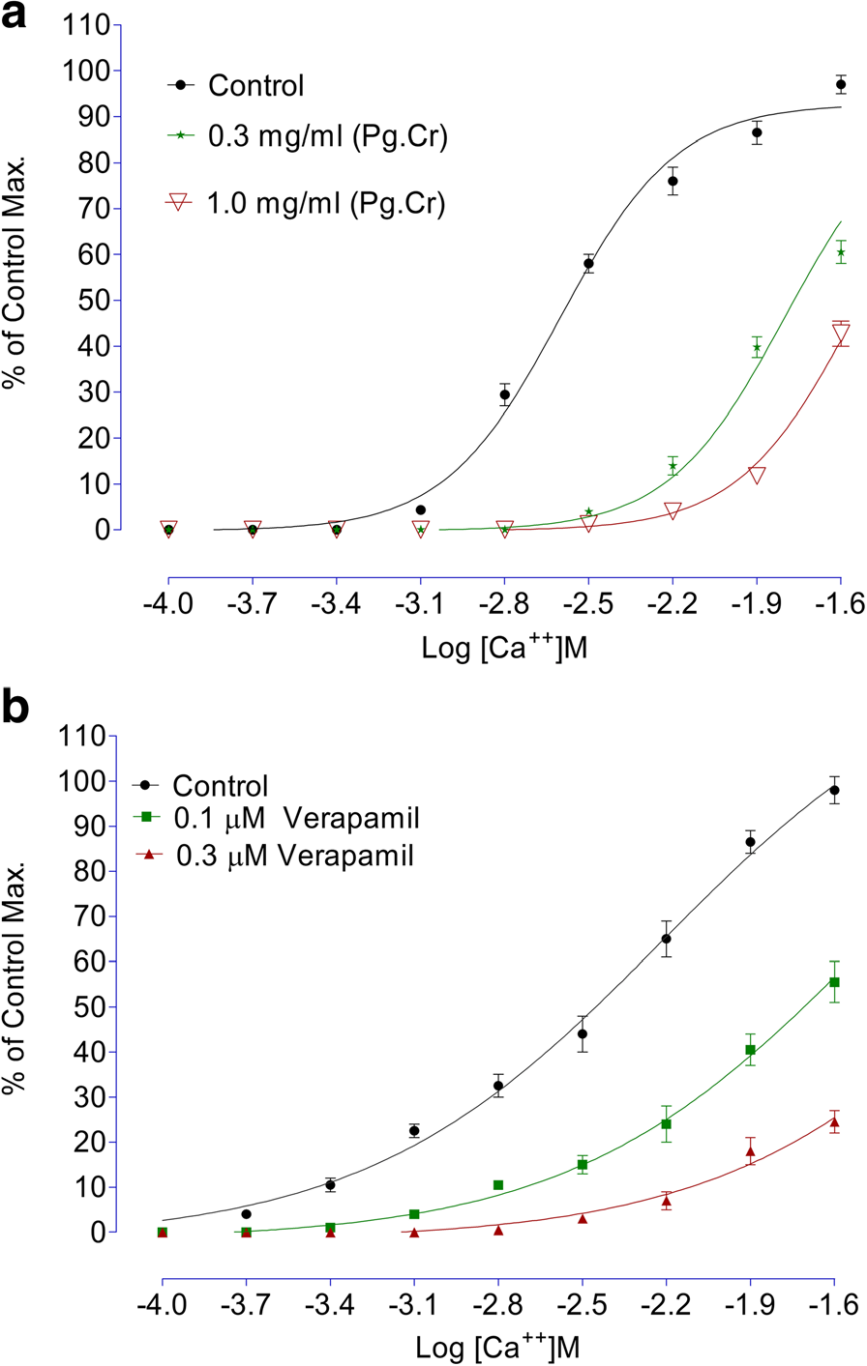
**a** Effects of *Punica granatum* on calcium chloride curves vs. respective control Maximum. **b** Effects of Verapamil on calcium chloride curves vs. respective control maximum

The effect of Pg. Cr on spontaneous rabbits’ jejunal preparations, in presence and absence of 0.03 μM atropine, are shown in Fig. 2. Maximum spasmogenic response (28 ± 1.5% of control maximum) was on 5 mg/ml. On 10 mg/ml, spasmolytic effect was observed in absence of atropine. However, in presence of atropine, whence the Pg. Cr was tried in similar concentrations, spasmogenic effect was blocked. Respective EC_50_ values in absence and presence of atropine are 9.7 ± 0.3 and 3.12 ± 0.45 mg/ml (Fig. 2). This left shift in EC_50_ values imply for the involvement of cholinergic receptors [25]. In addition to cholinergic receptors, GIT is rich with histaminergic receptors as well. So we tested Pg. Cr in presence of loratadine (0.02 and 0.08 μM). In the presence of 0.02 μM and 0.08 μM of loratadine HCl, respective EC_50_ are 5.6 ± 0.4 and 2.8 ± 0.15 mg/ml. Again left shift in the EC_50_ values suggest for the involvement of histaminergic receptors in the spasmogenic response. This suggests that loratadine blocked the spasmogenic response. Relaxant effect was observed in higher concentrations as it was in absence of atropine (Fig. 3). The involvement of histaminergic receptors in spasmogenic response may be beneficial, particularly, in indigestion where gastro-prokinetic response is sometimes required. However, the idea of gastro-prokinetic may be further explained by designing an in vivo model to study effects of Pg. Cr in low concentrations as our findings are totally different than the finding of Qnais et al. (2007) [13], where they have proven that the rind has spasmolytic activity. Perhaps relaxing effects reported by Qnais et al. (2007) may be due to high concentration of the rind extract as Pg. Cr relaxed the spontaneous activity in high concentration i.e., 10 mg/ml. In an attempt to explore the possible mode of action of relaxing effect, effects of Pg. Cr on KCl induced and Barium chloride induced contractions are shown in Fig. 4. EC_50_ for relaxant effects of Pg. Cr on KCl-induced contractions is 8.6 ± 1 mg/ml (*n* = 3). It is evident that KCl in high concentration opens the voltage gated calcium channels and thus promotes the depolarization of tissues. A positive relaxing effect on KCl induced contortions suggests for the involvement of voltage gated calcium channels [25, 27]. EC_50_ for effects on KCl induced contractions is 8.6 ± 1 mg/ml. However, to rule out the involvement of release of calcium from internal stores, effects of Pg. Cr on barium chloride induced contractions are also shown in Fig. 4. There is no effect of Pg. Cr on barium chloride induced contractions, which suggests the non-involvement of release of calcium from internal stores [26]. These relaxing effects also resemble the effects of verapamil on spontaneous as well as KCl-induced contractions with EC_50_ of 0.34 ± 0.02 μM and 0.035 ± 0.05 μM (Fig. 5), respectively. As verapamil is a standard calcium channel blocker that relaxed the spontaneous activity, hence, we used verapamil as standard drug for effects on CCRCs. For confirmation of inhibition of voltage gated calcium channels, effects of various concentrations of Pg. Cr on CCRCs are shown in Fig. 6a. Similarly, effects of verapamil on CCRCs are also shown in Fig. 6b. In the presence of 0.3 mg/ml of Pg. Cr, the curves gained maximum amplitude up to 70% of control maximum. While in concentration of 1.0 mg/ml, its amplitude could hardly reach to 40% of control maximum (Fig. 6a) suggesting for inhibition of voltage gated calcium channels. Voltage gated calcium channels play a vital role in the regulation of peristaltic movements of the intestine as it helps in periodic depolarization and repolarization of the muscles of gastrointestinal tract [18–22]. Since KCl induces contractions via calcium influx from extracellular medium to intracellular medium, hence, relaxing effects on KCl-induced contractions may be regarded to follow calcium channel blocking mechanisms of the voltage gated calcium channels [21]. Pg. Cr produced a right shift in the presence of 0.3 mg/ml with EC_50_ -1.8 ± 0.09 *vs.* control EC_50_ -2.6 ± 0.01 (log [Ca^++^]M). Similarly, EC_50_ for verapamil (0.1 μM) is −2.4 ± 0.011*vs.* Control EC_50_ -2.4 ± 0.01 (log [Ca^++^]M) (Fig. 6b). The right shift of Pg. Cr resembles the right shift of Verapamil. Hence, it is suggested that Pg. Cr may follow the inhibition of influx of calcium through voltage gated calcium channels. The relaxant activity may be attributed to the phytochemicals present in *Punica granatum,* particularly the flavonoids that have antispasmodic activity in general.

## Conclusion

Our findings suggest that rind of *Punica granatum* has spasmogenic as well as spasmolytic activity. Spasmogenic activity may follow the involvement of histaminergic and cholinergic receptors. However, spasmolytic activity may follow the inhibition of voltage gated calcium channels. The possibility of other mechanisms cannot be ruled out.
